# OpenWorm: an open-science approach to modeling *Caenorhabditis elegans*

**DOI:** 10.3389/fncom.2014.00137

**Published:** 2014-11-03

**Authors:** Balázs Szigeti, Padraig Gleeson, Michael Vella, Sergey Khayrulin, Andrey Palyanov, Jim Hokanson, Michael Currie, Matteo Cantarelli, Giovanni Idili, Stephen Larson

**Affiliations:** ^1^Neuroinformatics Doctoral Training Centre, University of EdinburghEdinburgh, UK; ^2^OpenWorm ProjectSan Diego, CA, USA; ^3^Department of Neuroscience, Physiology and Pharmacology, University College LondonLondon, UK; ^4^Department of Physiology, Development and Neuroscience, University of CambridgeCambridge, UK; ^5^Laboratory of Complex Systems Simulation, A.P. Ershov Institute of Informatics SystemsNovosibirsk, Russia; ^6^Department of Biomedical Engineering, Duke UniversityDurham, NC, USA

**Keywords:** integrative modeling, *C. elegans*, emergent behavior, complex systems simulation, open science

## Abstract

OpenWorm is an international collaboration with the aim of understanding how the behavior of *Caenorhabditis elegans* (*C. elegans*) emerges from its underlying physiological processes. The project has developed a modular simulation engine to create computational models of the worm. The modularity of the engine makes it possible to easily modify the model, incorporate new experimental data and test hypotheses. The modeling framework incorporates both biophysical neuronal simulations and a novel fluid-dynamics-based soft-tissue simulation for physical environment-body interactions. The project's open-science approach is aimed at overcoming the difficulties of integrative modeling within a traditional academic environment. In this article the rationale is presented for creating the OpenWorm collaboration, the tools and resources developed thus far are outlined and the unique challenges associated with the project are discussed.

## 1. Introduction

OpenWorm is an open science project dedicated to providing a flexible tool for *C. elegans* researchers to explore hypotheses of biological function *in silico*. Recently, studies from experimental neuroscience have called for such computational tools (Wen et al., 2012). The vision of the project is to build a modular and extensible simulation of the worm, initially focusing on its nervous system, that can be adapted to address specific scientific questions. The aim is not to produce a single model of *C. elegans*, but rather to construct a general simulation framework that enables the creation of a family of worm models. The different models can feature distinct neuronal and biomechanical modules or include new components capable of simulating broader aspects of biophysics.

*C. elegans* is a nematode that lives in soil environments where it searches for and consumes bacteria. It is one of the most studied multicellular organisms: its genome has been sequenced (*C. elegans* Sequencing Consortium, 1998), studies of its biology have resulted in three different Nobel prizes (Brenner, 2003; Mello, 2007; Chalfie, 2009), and it is currently the only organism that has its connectome diagram mapped (White, 1985; Varshney et al., 2011; Jarrell et al., 2012). The worm has also been the subject of many computational studies, most of which have aimed to understand its locomotion (Ferree and Lockery, 1999; Suzuki et al., 2005a,b; Boyle et al., 2008; Berri et al., 2009). Recent technological developments have also pushed forward the state of the art in experimental studies of the worm, such as the demonstration of dynamic “mind control” of *C. elegans* via direct laser stimulation of individual neurons expressing optogenetic components (Leifer et al., 2011) and simultaneous whole-animal 3D imaging of neuronal activity using light-field microscopy (Prevedel et al., 2014).

However, despite the copious amounts of data being obtained, modeling efforts and advanced experimental techniques, a comprehensive understanding of how the behavior of the worm emerges from the underlying physiological processes has not yet been achieved (Cohen and Sanders, 2014; Gjorgjieva et al., 2014). The complexity of mechanisms regulating behavior strongly argues for a holistic approach where results are integrated into a functioning computational simulation (Kitano, 2002; Palsson, 2002; Di Ventura et al., 2006).

Distinguished computer scientist David Harel called the task of creating a four-dimensional simulation of a biological system that is “true to all known facts” a grand challenge of computing (Harel, 2004). While building a *perfect* simulation of *C. elegans* is not feasible, nonetheless an integrative simulation based on what is currently known could help further define and choose between competing hypotheses, help generate new experimentally testable predictions, and expose gaps in our knowledge.

Due to its relative simplicity, *C. elegans* is an ideal candidate to help push the boundaries of *in silico* neuroscience. The OpenWorm project aims to help this cause by developing a common simulation platform for creating computational models of the organism. In addition to developing this resource for the community, a secondary ambition of the project is to gain a heuristic understanding of how the range of behaviors reproduced by the model scales with biological realism. The modular simulation engine will allow the user to substitute various models of biophysical processes. For example, neurons can be represented by single or multi-compartmental models. The modular structure opens up the possibility of studying how different simulation modules act together to bring forth the emergent information processing architecture of the total system. Exploring a family of models using different levels of abstraction could help to identify critical biophysical processes and hence advance a mechanistic understanding of how the behavior of *C. elegans* is generated.

## 2. Resources developed to date

**Figure 1:**
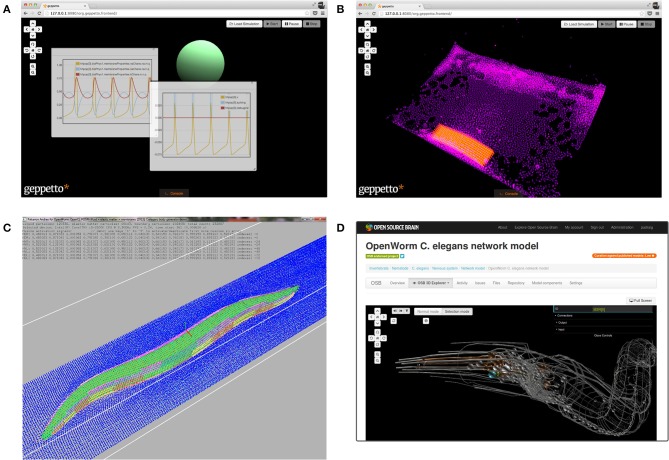
**Components which have been developed and made available by the OpenWorm initiative**. **(A)** Screenshot of the web interface of Geppetto, showing a single compartment neuron model (green sphere) with Hodgkin Huxley type ionic conductances. The floating windows show the time evolution of the cell membrane potential (central frame) and activation and inactivation variables of the ion channels (left frame). **(B)** Screenshot of a soft body physics simulation in Geppetto, showing a block of simulated muscle (orange) in a liquid environment (purple). **(C)** Screenshot of the Sibernetic application showing a simulated worm body (green) in a liquid environment (blue). This preliminary worm model reproduces the geometry of *C. elegans*, the model includes a hydrostatic skeleton with elastic impermeable shell and internal pressurized liquid. There are 95 body wall muscles which can receive signals from artificial neurons and contract, resulting in movement of the worm in its virtual world. **(D)** The online 3D visualization of the NeuroML translation of the 302 reconstructed neurons in *C. elegans*. The NeuroML files reside in an open source repository (https://github.com/openworm/CElegansNeuroML) and the Open Source Brain website can retrieve these to provide an in-browser visualization of the structure of the network without the need for installing any software on the user's machine.

To create a virtual worm, a software platform is required that is generic enough to enable multiple model components to be integrated together. To solve this problem, the OpenWorm team developed Geppetto (Idili et al., 2011; Cantarelli et al., 2014), a web-based simulation platform. It acts as middleware to mediate between different simulators. Currently Geppetto includes two major modules: one that simulates the electrical activity of the nervous system and a soft body physics module which will be used to calculate the interaction of the worm with its environment (Figures 1A,B). The integration of these two modules will make it possible to simulate the contraction of muscle tissue in response to the electrical stimuli generated by the nervous system (Palyanov et al., 2012).

The Sibernetic physics engine (Palyanov et al., 2013) was developed by the OpenWorm team in parallel to Geppetto to simulate the biomechanics of soft tissues and the environment of the worm (Figure 1C). It can handle the simulation of liquids, elastic matter and solids with various physical properties. Sibernetic is based on the predictive-corrective incompressible smoothed particle hydrodynamics (PCISPH) algorithm (Solenthaler and Pajarola, 2009) with modifications to incorporate boundary-handling as proposed by Ihmsen et al. (2010) and a surface tension model based on Becker and Teschner (2007). The advanced features of Sibernetic are currently being incorporated into the soft body physics simulation module of Geppetto. Sibernetic, to the best of our knowledge, is the first open source, parallel OpenCL/C++ PCISPH high-performance physics engine.

To create a model of the nervous system, the OpenWorm project has incorporated data from the *C. elegans* connectome (Varshney et al., 2011) and the 3D anatomical map of the body plan (Grove and Sternberg, 2011). The NeuroML language (Gleeson et al., 2010) was used to encode the multi-compartmental structure of each of the 302 neurons, along with information on the locations and types of chemical and electrical synapses known to exist. The NeuroML *C. elegans* connectome can be viewed in 3D on the Open Source Brain website (Figure 1D) and this model can also be visualized using the graphical application neuroConstruct (Gleeson et al., 2007). The spatial connectome can be exported to the NEURON simulator (Carnevale and Hines, 2006) to compute the electrical activity of the system. However, the model does not yet incorporate realistic active membrane conductances. To allow Geppetto to run models in NeuroML format, a module which wraps the jLEMS simulator has been developed. jLEMS is the Java based reference implementation of the Low Entropy Model Specification language, the language on which the latest version of NeuroML is built (Cannon et al., 2014).

While Geppetto is in the early stages of development, the current implementation can already handle some structural features of *C. elegans* nervous system such as the organism's connectome. Numerous physiological processes and characteristics are neglected in the current framework, for example cell-specific ion channel distribution and kinetics. Geppetto was constructed to handle these limitations. What is currently unknown can be incorporated into the simulation when the appropriate data becomes available or estimated parameters can be used if testing a specific hypothesis requires their presence. Detailed information about the OpenWorm resources can be found on the project's documentation website (OpenWorm, 2014).

## 3. Modeling complex systems—the scientific challenge

There are two main challenges in creating an *in silico C. elegans*. First, the inherently multiscale nature of biological systems and therefore the lack of a single level of abstraction at which to model physiological processes (Voit, 2013). The second challenge is the interconnected physiology of the organism and as a consequence the large number of mechanisms that must be represented in single model (Kaneko, 2006; Bargmann, 2012). This section illustrates how these two characteristics of living organisms creates limitations for their virtual counterparts.

Computational models in neuroscience often model a system at a particular scale, such as cellular or network level. In the integrative framework of OpenWorm there is no single preferred scale, and so Geppetto has been developed as a multiscale platform (Vlachos, 2005; Weinan, 2011). There is the lack of understanding about the level of detail at which the different constituents of *C. elegans* nervous system—neurons, synapses, ion channels etc.—must be modeled to preserve the emergent behavior of the worm. Consider neural morphology. Subcellular calcium signals in the RIA interneurons encode head movement (Hendricks et al., 2012). Therefore, subcellular calcium dynamics have a functional consequence for these neurons, but it may not be the case for every neuron. The advantage of OpenWorm's modular engine is that it allows to represent the different components of the nervous system with different level of detail. This feature allows a combinatorial exploration of how the nervous system's components collectively bring forth the emergent macroscopic behavior. Hence OpenWorm can further a mechanistic understanding of how and which microscopic processes lead to macroscopic consequences.

The interconnectedness of biological systems is reflected by the numerous mechanisms regulating the behavior of the worm. Neurons communicate with electrical signals, but it is often overlooked that the nervous system itself is awash in a chemical environment that interacts with the electrical signaling processes (Bargmann, 2012; Cohen and Sanders, 2014). For example, *C. elegans* adjusts its rate of locomotion in the presence of food (Sawin et al., 2000). This behavioral adaptation is achieved through a dopaminergic neural circuit (Sawin et al., 2000; Omura et al., 2012). Moreover *C. elegans* expresses over 250 different neuropeptides, but for most of them little is known about how they affect the physiology of neurons and other cells (Li and Kim, 2008).

The multiscale nature of biological systems and their interconnectedness will inevitably place limitations on any computational model of *C. elegans*. For example consider the speed of locomotion adjustment discussed earlier. The current Geppetto simulations do not include dopamine signaling hence the models will not reproduce this aspect of locomotion. While this is an expected restriction on the validity domain of the first OpenWorm models, the extensibility of Geppetto means this restriction need not be permanent.

## 4. Evaluating models of the worm

### 4.1. The movement and physiological testing engine

OpenWorm is developing a simulation framework that enables the creation of a family of worm models. Each model created by different groups should be evaluated with respect to the original purpose of the research. To provide help for the evaluation process the project will feature a movement and a physiological testing tool, these will enable the scientific community to evaluate their hypothesis directly on OpenWorm models. In return the test results will enable the OpenWorm team to better integrate recent results and therefore allow our development to be data-driven.

OpenWorm's efforts are focused not on modeling every physiological process of the nervous system, but rather on capturing their function. The externally observable behavior, or “macroscopic behavior” as it will be referred to, is the end product of all the nervous system's activity. Macroscopic behavior is the aggregated, high level output of all the underlying physiological processes. Therefore, to a close approximation the function of the nervous system is to generate macroscopic behavior. If a model fails to reproduce some arbitrary processes, but it reproduces the worm's macroscopic behavior, then the important aspects of the nervous system's function are still captured.

To assess how well an OpenWorm model reproduces the macroscopic behavior of *C. elegans*, a movement validation engine is being developed. It is as an automated test based on quantifiable aspects of behavior and was inspired by the techniques used to discriminate the behavioral phenotypes of mutant worms (Yemini et al., 2013). The recent *C. elegans* behavioral database (Yemini et al., 2013) provides an unmatched source of information about the macroscopic behavior of both wild type and mutant worm strains. For each strain the database contains a large number of measurables such as speed of locomotion, frequency of reversing direction, of omega turns, etc. The worm's behavior should be studied under a wide array of stimulus conditions, however the *C. elegans* behavioral database only contains recordings of worms browsing in a bacterial layer. This is currently a limitation, but as data becomes available about behavior in other stimulus conditions, the worm's actions could be re-analyzed using the same tools.

The aim of the OpenWorm project is not to model the behavior of the worm directly, but to understand how macroscopic behavior emerges through the underlying physiological processes. Therefore, building a model that behaves in an identical manner to *C. elegans* is necessary, but not sufficient. Traditionally, artificial intelligence research has attempted to reproduce human-like intelligence without simulating the physiological processes of the brain. Similarly, one can attempt to directly model the behavior of the worm without the underlying biological elements. However, such a model, even if reproduces the macroscopic behavior of the worm would provide limited scientific value. Biologists would be unable to relate the measurements they make in the lab to variables in the simulation.

OpenWorm simulates the biology of the worm, hence the virtual physiology of the model can be examined. Geppetto allows the extraction of time series of physiological variables, such as membrane potentials, ionic concentrations, body wall forces etc. This feature will contribute to OpenWorm's usability in *C. elegans* laboratories. Just like modifications to cars are analyzed in computer-aided design (CAD) programs before being tested on the road, scientists could make perturbations *in silico* before beginning the expensive and time-consuming *in vivo* experiments. Conversely, having scientist users will engender a feedback process that will make the development of OpenWorm data-driven, helping to improve the models in the first place.

### 4.2. The imitation game approach

As stated in the Introduction a secondary ambition of the project is to explore heuristically how the complexity of behaviors reproduced by the models scales with biological realism. When evaluating OpenWorm models for this objective Harel has argued for a Turing-like test (Harel, 2005). This framework does not replace the testing units discussed in the previous subsection, but provides an alternative point of view.

According to the Popperian criteria of empirical falsification (Popper, 1968), a scientific theory must make falsifiable predictions and the value of a theory lies in how these forecasts compare against observations. For example general relativity makes clear predictions about the orbit of Mercury. In this case it is straightforward to compare the observation and the prediction, because both has a clear mathematical form. In contrast there is no unambiguous definition for many complex biological phenomena. For example there is no universally accepted definition for what is life or human intelligence (Neisser et al., 1996; McKay, 2004; Schlinger, 2012). For these systems the “imitation game” has been proposed as a means to evaluate models mimicking the phenomena (Turing, 1950; Cronin et al., 2006).

The most famous example of an imitation game is the Turing test. There is no universally accepted definition of human intelligence against which the behavior of a machine could be measured. This problem appeared in the early days of artificial intelligence research and motivated Turing to invent his test. Turing argued that if a human interrogator is unable to distinguish between a human and a computer through a text-only communication channel, then the computer can be considered intelligent (Turing, 1950). The Turing test overcomes the lack of a universally accepted definition for intelligence by replacing the comparison of measured and predicted data with the requirement for indistinguishability by an expert agent. As a result the Turing test finds an operational way to assess whether a machine can think regardless of any definition of intelligence (Cronin et al., 2006).

In common with intelligence, there is no unambiguous definition of *C. elegans* behavior. There is no quantitative assessment that captures the totality of *C. elegans* behavior under all the possible environmental and stimulus conditions.

**Figure 2:**
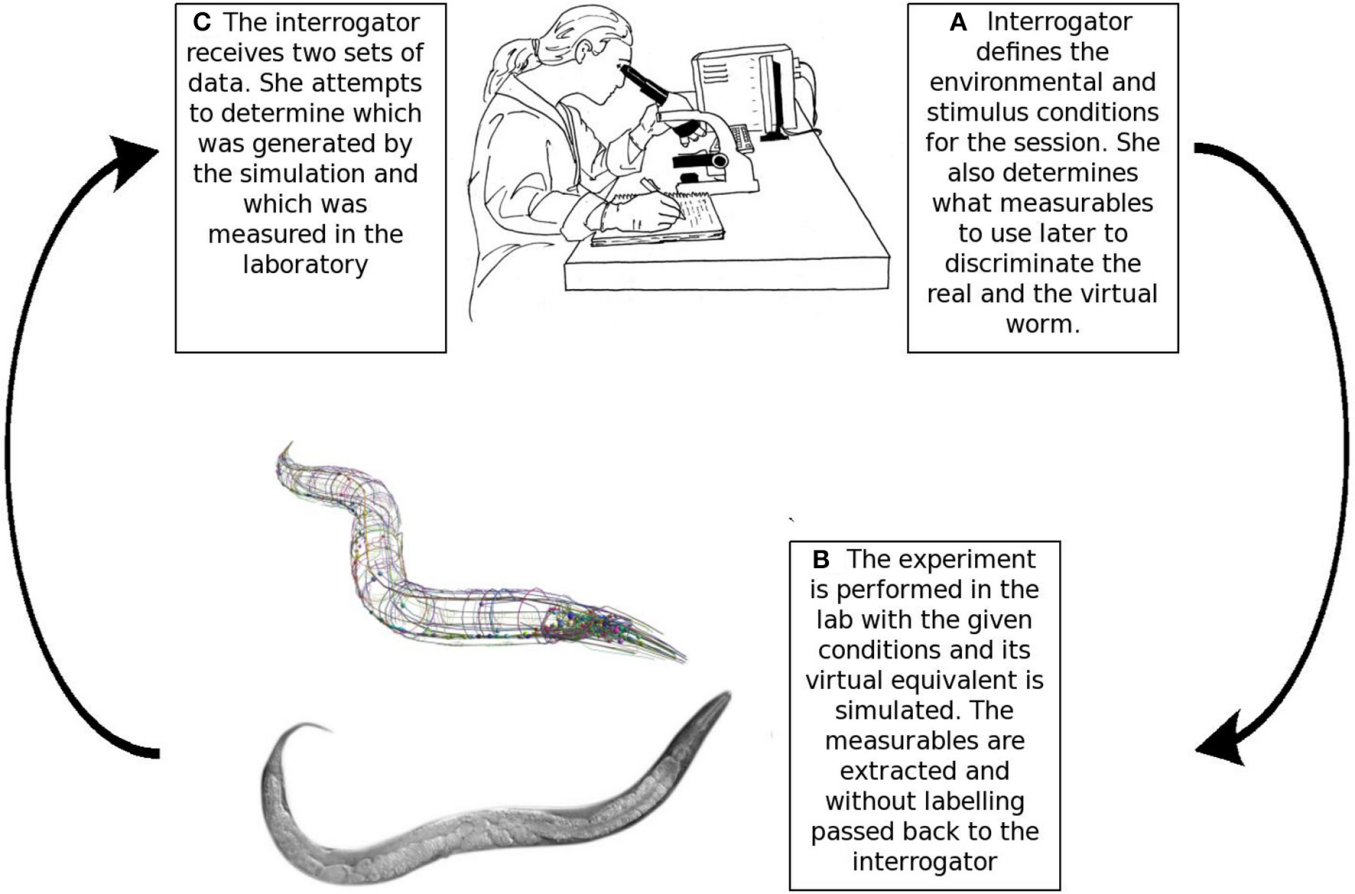
**The Turing-like test for *in silico* biology**. **(A)** The interrogator determines the experimental and stimulus conditions for the experiment. She also chooses the target measurables that will be later used to distinguish the real and simulated organisms. **(B)** The experiment and its virtual equivalent are conducted and the measurables for both the real and virtual worm are passed to the expert without labels. **(C)** The interrogator attempts to identify the data generated *in silico*. She is allowed to use her intuition or any statistical data analysis methods. If the expert can not reliably distinguish between the real and simulated organisms based on objective measurables, then the model passed the test for the given conditions. A future understanding of the worm could realize new ways to discriminate the real and the simulated worms. Therefore, the Turing test's results are not absolute, but evolve with time and knowledge (image of N2 adult *C. elegans* is courtesy of M. Boxem, top drawing of the scientist is courtesy of G.P. Ferenczi).

Harel argued that models of biological systems could be validated through a Turing-like test, he developed this idea specifically in the context of a four-dimensional simulation of *C. elegans* (Harel, 2005). The core idea of indistinguishability by an expert can be extended to models of systems biology. Instead of text conversations, the interrogator attempts to distinguish the real and simulated organisms based on objective experimental measurables. Harel reasoned that an expert's inability to differentiate between the real and simulated organism based on objective measurables indicates a “complete model” (Harel, 2004, 2005). Note that the imitation game does not provide absolute verification. Whether a model passes the imitation game depends on the current state of knowledge, a model can become falsifiable in the future due to a better understanding of the worm (Harel, 2005). The test process is illustrated in Figure 2.

The Turing test can be conducted in various environmental and stimulus conditions. The richer the environment and the more stimuli the worm encounters during a session, the stronger the test will be. The scale of possible environmental complexity ranges from placing a worm on a plain Petri dish to three-dimensional soil environments like the natural habitat of the worm. The *C. elegans* behavioral database contains recordings of worms crawling in a bacterial layer, therefore this data will alone not suffice to fully verify an OpenWorm model. Similarly to environmental complexity, the validity domain of a model can be examined in time. Eventually the model could be tested for its ability to reproduce the sleep-like states (Raizen et al., 2008; Cho and Sternberg, 2014) and the learning capabilities (Ardiel and Rankin, 2010) of a real worm.

The Turing test is a subject of debate and there are ongoing discussion about its value as a scientific tool (Copeland, 2003; Saygin et al., 2003; LaCurts, 2011; Grosz, 2012; Berrar et al., 2013). Regardless of the interpretational issues, the imitation game provides a conceptual tool to think about model verification. OpenWorm does not plan to run the imitation game, but would provide technical support if any group wishes to put the virtual worm to the test in this manner.

## 5. Openworm's approach to open science

The sociological roadblocks of the modern scientific enterprise are major obstacles to integrative modeling. For example, inability to pool data together using common standards results in the creation of separated silos of data (Martone et al., 2004; Akil et al., 2011). This data fragmentation has led to a series of statistically underpowered studies in neuroscience (Button et al., 2013). Reasons for this state of affairs include lax requirements for releasing data and methods out into the public sphere, and for sharing source code. As a consequence, there is a mismatch between the vast amount of data being collected as part of the scientific process, and that which is available for incorporating into and for testing models.

Solutions have been proposed to ameliorate these problems. These include the improved use of informatics (Roysam et al., 2009; Akil et al., 2011), an increased emphasis on the sharing of data and methods, as well as the use of collaboration to improve the reproducibility and statistical power of experimental results (Button et al., 2013). A more radical proposal has been that of open science (Nielsen, 2012). Open science involves the use of online tools to share scientific knowledge, leveraging the untapped potential of individuals distributed around the world to solve scientific problems. Reusability is a first priority via an online, common and shared repository of information.

To overcome these roadblocks for integrative modeling, OpenWorm began as an open-science project. The project reports its intermediate results via public discussions on the web, provides a roadmap of its activities which is open to the public, uses open mailing lists and public online chats to discuss the project and makes all code accessible on the web. Through the use of these techniques, the project seeks to achieve a higher degree of community engagement and a paper trail that improves the reproducibility of results. The project is not centralized, everything is done by dedicated individuals from different parts of the world whose research overlap with the goals of OpenWorm.

OpenWorm continues to seek out mutually beneficial partnerships with individuals or laboratories. Developing a virtual *C. elegans* is an ambitious scientific enterprise and both data and further expertise are needed. There is a great amount of work to be done and success can only be achieved with a community effort.

### Conflict of interest statement

The authors declare that the research was conducted in the absence of any commercial or financial relationships that could be construed as a potential conflict of interest.
